# Dose escalation can maximize therapeutic potential of sunitinib in patients with metastatic renal cell carcinoma

**DOI:** 10.1186/s12885-018-4209-9

**Published:** 2018-03-15

**Authors:** Anikó Maráz, Adrienn Cserháti, Gabriella Uhercsák, Éva Szilágyi, Zoltán Varga, János Révész, Renáta Kószó, Linda Varga, Zsuzsanna Kahán

**Affiliations:** 1 Department of Oncotherapy, University of Szeged, Korányi fasor 12, Szeged, H-6720 Hungary; 2 Institute of Radiotherapy and Clinical Oncology, Borsod County Hospital and University Academic Hospital, Szentpéteri Kapu 72-76, Miskolc, H-3526 Hungary

**Keywords:** Metastatic renal cell cancer, Sunitinib, Dose escalation, Improved outcome, Toxicity

## Abstract

**Background:**

In patients with metastatic renal cell cancer, based on limited evidence, increased sunitinib exposure is associated with better outcome. The survival and toxicity data of patients receiving individualized dose escalated sunitinib therapy as compared to standard management were analyzed in this study.

**Methods:**

From July 2013, the data of metastatic renal cell cancer patients with slight progression but still a stable disease according to RECIST 1.1 criteria treated with an escalated dose of sunitinib (first level: 62.5 mg/day in 4/2 or 2 × 2/1 scheme, second level: 75 mg/day in 4/2 or 2 × 2/1 scheme) were collected prospectively. Regarding characteristics, outcome, and toxicity data, an explorative retrospective analysis of the register was carried out, comparing treatments after and before July 1, 2013 in the study (selected patients for escalated dose) and control (standard dose) groups, respectively.

**Results:**

The study involved 103 patients receiving sunitinib therapy with a median overall and progression free survival of 25.36 ± 2.62 and 14.2 ± 3.22 months, respectively. Slight progression was detected in 48.5% of them. First and second-level dose escalation were indicated in 18.2% and 4.1% of patients, respectively. The dosing scheme was modified in 22.2%. The median progression free survival (39.7 ± 5.1 vs 14.2 ± 1.3 months (*p* = 0.037)) and the overall survival (57.5 ± 10.7 vs 27.9 ± 2.5 months (*p* = 0.044)) were significantly better in the study group (with dose escalation) than in the control group. Patients with nephrectomy and lower Memorial Sloan Kettering Cancer Center (MSKCC) scores showed more favorable outcomes. After dose escalation, the most common adverse events were worsening or development of fatigue, hypertension, stomatitis, and weight loss of over 10%.

**Conclusions:**

Escalation of sunitinib dosing in selected patients with metastatic renal cell cancer, especially in case of slight progression, based on tolerable toxicity is safe and improves outcome. Dose escalation in 12.5 mg steps may be recommended for properly educated patients.

## Background

Sunitinib malate, an oral multi-targeted tyrosine kinase inhibitor (TKI) is considered to be one of the standard first-line therapeutic options in metastatic renal cell cancer (mRCC) [1]. It is a small molecule indolinone [2] which binds directly to the kinase domain of receptor tyrosine kinases (RTKs) within an adenosine triphosphate (ATP) binding pocket between two lobes of the KIT kinase domain, preventing phosphorylation and activation [3–5]. It selectively targets RTKs, which are important in RCC. Sunitinib has direct anti-tumor effects via binding the unactivated conformation of KIT and via platelet-derived growth factor receptor alpha polypeptide (PDGFRA) inhibition. The dual inhibitor activity against vascular endothelial growth factor receptors 1 and 3 (VEGFR 1 and VEGFR3), and platelet-derived growth factor receptor beta polypeptide (PDGFRB) on endothelial and pericyte membranes enhances anti-angiogenesis [6].

Sunitinib has been approved by the regulatory authorities after it had been demonstrated to improve progression-free survival (PFS), overall survival (OS), objective response rate (ORR), and quality of life compared with interferon-alpha in previously untreated metastatic RCC patients [1, 7–9]. According to the international guidelines (e.g., NCCN, ESMO, EAU), sunitinib can be used as first-line treatment in patients with advanced or metastatic dominantly clear cell histological type RCC whose condition has good or intermediate prognosis [10–12]. Sunitinib has become the gold standard first-line therapy of mRCC in the past decade, and it has been used worldwide in this patient population in wider indications as well [10–16].

The therapeutic administration of sunitinib and the dedicated patient population for this drug would be changing and would be refined in the near future. The preliminary results of the presented Checkmate-214 phase 3 trial with respect to mRCC, in which sunitinib was the comparator of the investigated drugs [17], the survival rates were more favorable in case of the immune checkpoint inhibitor nivolumab and ipilimumab combination compared to sunitinib administered alone, in poor and intermediate risk groups.

The standard treatment schedule of sunitinib is 50 mg for 28 days with a 14-day break [13–15]. Alternate scheduling (2 weeks on/1 week off) can also be used to manage toxicity, but currently no robust data are available supporting it [16]. The dose can be adjusted according to the patient’s response to the treatment, but it should be kept within the range of 25 to 75 mg [18]. At higher sunitinib doses, the direct anti-cancer effect of the drug may be predominant.

Despite the efficacy of sunitinib therapy, the condition of initially responding patients may progress due to the acquired resistance. The underlying mechanisms for that may be the continuous VEGF axis activation via upstream or downstream effectors [19–22], b-fibroblast growth factor (bFGF), c-met, interleukin-8 (IL-8), and angiogenic cytokine pathways [23], altered pharmacokinetics, drug sequestration [24], and epithelial to mesenchymal transition [25]. Drug resistance is associated with a transient increase in tumor vasculature and epigenetic changes in histone proteins in the chromatin, which contribute to tumor angiogenesis by inactivating the anti-angiogenic factors [26]. However, the drug-induced resistance can be overcome by sunitinib dose escalation [26]. If patients tolerate the standard regimen, the increased sunitinib exposure is associated with longer PFS, OS, and a higher response rate [27, 28].

The aim of our study was to analyze the maximal efficiency and the side-effects of escalated dose sunitinib for metastatic RCC in the everyday practice.

## Methods

### Patients

An explorative retrospective analysis of a prospective mRCC register was carried out at the Department of Oncotherapy University of Szeged, Hungary. 103 patients with MSKCC (Memorial Sloan-Kettering Cancer Center) good (0 unfavorable factor) or intermediate risk (1 or 2 from the following 5 unfavorable factors: 1. time from diagnosis to systemic treatment < 1 year; 2. hemoglobin < lower limit of normal level; 3. calcium > 10 mg/dL or 2.5 mmol/L; 4. LDH > 1.5 x upper limit of normal; 5. Karnofsky performance status < 80%) [1, 18] were treated with sunitinib between January 2010 and December 2016. The study was performed in accordance with the Hungarian and the EU drug law and relevant medical and financial guidelines of the Hungarian health authorities. The study was approved by the regional ethics committee (registration number WHO 3482/2014).

The patients received first-line sunitinib after having undergone nephrectomy or kidney biopsy and embolization if nephrectomy was not feasible. Histological and staging examinations, such as abdominal and chest CT (and bone scintigraphy and skull CT if clinically indicated), were performed before initiating the therapy.

### Sunitinib therapy and dose modifications

**Fig. 1:**
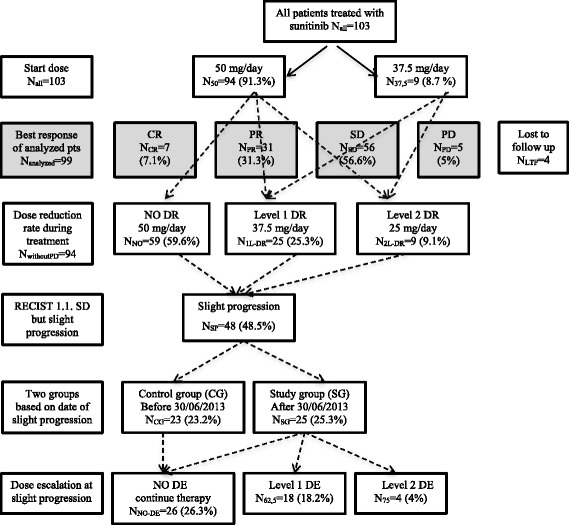
Flowchart of sunitinib dose modifications. (CG – control group, CR – complete remission, DE – dose escalation, DR – dose reduction, LTF – lost to follow-up, N – number of analyzed patients, PD – progressive disease, PR – partial remission, RECIST – Response Evaluation Criteria In Solid Tumors, SD – stable disease, SG – study group)

Patients received sunitinib monotherapy orally, in six-week cycles, at a dose of 50 mg once a day for 4 weeks, followed by a two-week rest period (4/2 scheme) in 94 (91.3%) cases. In 9 (8.7%) cases with advanced age and concomitant diseases, the therapy was started with a reduced dose of 37.5 mg. Physical and laboratory examinations were performed 2 to 4 weeks after the initiation of sunitinib therapy, and once every 6 weeks thereafter, while imaging examination, cardiac and thyroid gland function follow-ups were performed every 12 weeks. Adequate supportive therapy and proactive management of side-effects were applied. Dose reduction (DR), modification of dose scheme (DSM) (2 weeks on/1 week off), or therapeutic delay occurred due to the following reasons: grade 3/4 thrombocytopenia, neutropenia, hand–foot syndrome affecting walking, stomatitis or diarrhea of grade 3/4, which significantly influenced the nutrition or resulted in > 10% weight loss, hypertension of grade 3/4 developing despite being on combined antihypertensive therapy. The severity of adverse events was graded according to the National Cancer Institute Common Terminology Criteria for Adverse Events Version 4.0 (NCI CTCAE v4.0) [29]. The general condition of the patients was assessed according to the Karnofsky scale [30]. PFS and OS were defined from the onset of the medical treatment to the date of progression based on RECIST 1.1 or death, respectively. The evaluation of tumor response was performed every 12 weeks according to Response Evaluation Criteria In Solid Tumors (RECIST) 1.1. Sunitinib therapy was discontinued in case of progression per the RECIST criteria in all cases (compared to best response). If the CT indicated slight progression (SP) but still corresponded to stable disease according to the RECIST 1.1 criteria [31] in patients enrolled in the study after June 30, 2013 (study group), a dose escalation (DE) strategy was started with careful follow-up if any clinically significant side effect was detected. The dose was elevated first to 62.5 mg, and if a slight progression was still present or occurred again, to a level of 75 mg. Patients showing SP before the date of June 30, 2013 were enrolled in the control group (Fig. 1).

### Evaluation of the effect of dose escalation

The effects of dose escalation was analyzed on PFS and OS of both the entire patient population and the patients showing SP. Two groups of patients with SP were distinguished considering that the SP occurred before or after June 30, 2013; patients before that date were treated with an unchanged standard dose, despite the presence of SP. After that date, in cases without relevant side effects, a DE strategy was applied. The outcome was analyzed according to the characteristics of the patients of the two groups as well as the side effects and other factors that could influence the escalation of the dose.

### Statistical analysis

The association between PFS, OS and age, and the number of metastatic organs was analyzed using COX regression. The influence of the therapy-related factors (dose escalation, dose reduction, therapeutic lines after sunitinib, nephrectomy, and treatment group), and patient-related factors (gender, MSKCC score) on PFS and OS was analyzed with Kaplan–Meier analysis. To compare the median follow up times between control and study groups, the Mann-Whitney U Test was used. To determine the differences between the control and study groups, independent sample t-test and chi-square test were used for the continuous and categorical variables, respectively. To detect the independent role of nephrectomy and DE on the outcome, multivariate COX regression was used. All statistical analyses were performed by using SPSS 20.0 for Windows (SPSS Inc., Chicago, IL, USA).

## Results

### Patient characteristics

Out of the 103 patients who participated in the study, 80 (77.7%) were men and 23 (22.3%) were women (Table 1). The mean ± standard error (±SE) age was 62.27 ± 0.9 (range, 32–80) years, and 84.5% of the patients had undergone nephrectomy. The mean (±SE) MSKCC score was 1.7 ± 0.05, and the mean number of metastatic sites was 2.32 ± 0.11 (range, 1–5). Lungs, bone and distant lymph nodes were the most frequent localizations of metastases (Table 1). 68% of the patients had a comorbidity that required treatment. Hypertension, other cardiovascular disorders, and diabetes were the most common diseases. Hyperthyroidism and well-managed hypertension at the beginning of the therapy occurred in 5 (4.9%) and 32 (31.1%) patients, respectively. The rate of secondary tumors was relatively high (8.7%) as well as the rate of primary bone metastasis (45.6%). Mean ± SE value of baseline LVEF was 61.7 ± 3.2%. The histological type of the tumors was mainly clear cell renal cell cancer (ccRCC) in case of all patients, and in most cases pure ccRCC. No rare variants could be detected, but only sarcomatoid, papillary and chromophobe morphologies, and transformations in the ccRCC were present**.** No genetic analyses were performed to prove the familial origin of the renal cancer. The baseline characteristics of the patients are presented in Table 1.

**Table 1:** Baseline demographics of all patients and of patients with slight progression

Patients	N_all_ = 103		N_SP_ = 48	
Mean age, years ± SE	62.27 ± 0.9		61.76 ± 1.62	
Age range, years	32–80			
MSKCC score, mean ± SE	1.7 ± 0.05		1.6 ± 0.1	
Gender				
Male	80 (77.7%)		39	81.3%
Female	23	23 (22.3%)	9	18.7%
Number of patients after nephrectomy	87	84.5%	42	87.5%
Comorbidities				
Hypertension	32	31.1%	9	18.8%
Other cardiovascular disorders	12	11.6%	5	10.4%
Diabetes	11	10.7%	4	8.3%
Secondary tumors	9	8.7%	1	2%
Hyperthyroidism	5	4.9%	0	0%
Hematological disease	3	2.9%	0	0%
Psoriasis	2	1.9%	0	0%
Metastases				
*Mean number of metastatic sites (range)*	2.32 ± 0.11 (1–5)		1.79 ± 0.1 (1–3)	
*Location of metastases*				
Lungs	84	81.6%	39	81.2%
Bone	47	45.6%	16	33.3%
Distant lymph node	36	34.9%	20	41.7%
Liver	19	18.4%	7	14.6%
Brain	11	10.7%	0	0%
Suprarenal gland	9	8.7%	4	8.3%
Other (peritoneum, pleura, pancreas, local relapse, contralateral kidney, or thyroid gland)	–	‹8%	–	‹4%
Patients with synchronous metastases	94	91.2%	45	93.8%
Histopathological types			n	%
Purely clear cell renal cell type (ccRCC)	91	88.3%	46	95.8%
ccRCC with sarcomatoid morphology	7	6.8%	1	2%
ccRCC with papillary−/chromophobe−/ both	3 / 2 / 1	2.9 / 1.9 / 1.0%	1/0/0	2/0/0%

*ccRCC* clear cell renal cell cancer, *MSKCC* Memorial Sloan Kettering Cancer Center, *n* number of involved patients, *N* number of analyzed patients, *SE* standard error

### Sunitinib dose parameters and efficiency

No dose reduction (DR) had to be applied in 59 (59.6%) patients (50 mg/day in 4/2 or 2 × 2/1 scheme or 37.5 mg daily dose administered continuously in 2 cases). First-level (37.5 mg/day in 4/2 or 2 × 2/1 scheme) and second-level (25 mg daily dose in 4/2 or 2 × 2/1 scheme) dose reductions were required during the treatment in 25 (25.3%) and 9 (9.1%) cases, respectively. Sunitinib therapy had to be ultimately ceased within 12 weeks in 5 (5%) patients due to progression of the disease. The follow-up of four patients was incomplete; thus, their data were excluded from the final analyses.

The dosing scheme was modified (DSM) in case of 22 (22.2%) patients. A cycle delay of more than 7 days was needed in 15 (15.1%) patients because of an infection, herniotomy, dental intervention, diarrhea, neutropenia, or cardiac decompensation. Mean ± SE duration of the delay was 7.8 ± 3.3 days. The median PFS ± SE was 14.2 ± 3.22 (95% CI 7.87–20.52) months. Complete remission as the most favorable tumor response was achieved in 7 (7.1%) cases. Partial remission and stable disease were accomplished in 31 (31.3%) and 56 (56.6%) patients, respectively.

In cases of SP, the result of radiological revision according to RECIST 1.1 was stable disease in 48 (48.5%) cases. First-level (62.5 mg/day in 4/2 or 2 × 2/1 scheme) and second-level (75 mg daily dose in 4/2 or 2 × 2/1 scheme) dose escalations were indicated in 18 (18.2%) and 4 (4.1%) patients, respectively. The median ± SE duration of sunitinib therapy was 19.45 ± 2.01 (95%CI 14.87–22.94) months until definition of slight progression and 7.8 ± 1.55 (95%CI 4.74–10.85) months from date of SP to progression. The median OS was 25.36 ± 2.62 (95% CI 20.23–30.5), and the median follow-up time was 24.37 (1.33–93.83) months, respectively. Sunitinib therapy is still continued in 10 (10.1%) patients, and 5 patients underwent metastasectomy; their sunitinib therapy was discontinued and rechallenged in 3 (3%) of them. After progression on sunitinib therapy, no further therapy was administered in 30 (30.3%) cases, while in 47 (47.4%) and 5 (5.1%) patients, one and two therapy lines were applied, respectively.

### Factors influencing efficacy

PFS and OS were not influenced by the patients’ age, gender, the number/type of metastatic organ systems, and dose reduction in the overall population. Patients with nephrectomy and lower MSKCC scores showed more favorable outcomes in the studied population (Table 2).

**Table 2:** Factors influencing the outcome of sunitinib therapy in all patients

Specifications of analyzed patients *N* = 99	PFS-HR (95% CI)	*p*	OS-HR (95% CI)	***p***
Age	1.012 (0.987–1.038)	0.351	1.007 (0.981–1.035)	0.590
Number of metastatic organs	1.083 (0.891–1.317)	0.423	1.100 (0.896–1.350)	0.364
	PFS-HR (95% CI)	*p*	OS-HR (95% CI)	***p***
Gender				
man/ woman	1 / 1.367 (0.807–2.316)	0.245	1 / 1.388 (0.792–2.435)	0.252
MSKCC score				
0 / 1 / 2	1 / 3.770 (1.345–28.435) / 6.693 (1.813–49.061)	**0.019**	1 / 2.692 (1.355–20.445) / 5.199 (1.713–37.929)	**0.023**
Dose reduction				
Yes / No	1 / 1.492 (0.947–2.506)	0.065	1 / 1.553 (0.963–2.504)	0.071
Nephrectomy				
Yes / No	1 / 2.702 (1.508–4.840)	**0.001**	1 / 3.189 (1.741–5.842)	**< 0.001**
Dose escalation				
Yes / No	1 / 2.665 (1.486–4.780)	**0.001**	1 / 3.157 (1.613–6.179)	**0.001**
Dose scheme modification				
Yes / No	1 / 2.569 (1.437–4.595)	**0.001**	1 / 2.444 (1.288–4.636)	**0.006**
Therapeutic lines after sunitinib				
2 / 1 / 0	NA	NA	1 / 7.731 (2.318–25.787) / 4.043 (1.228–13.311)	**0.001**

Bold *p*-values are significant ‹0.05, *HR* hazard ratio, *MSKCC* Memorial Sloan Kettering Cancer Center, *mOS* median overall survival, *mPFS* median progression-free survival, *NA* not applicable, *OS* overall survival, *p p*-value, *PFS* progression-free survival, *SE* standard error

**Fig. 2:**
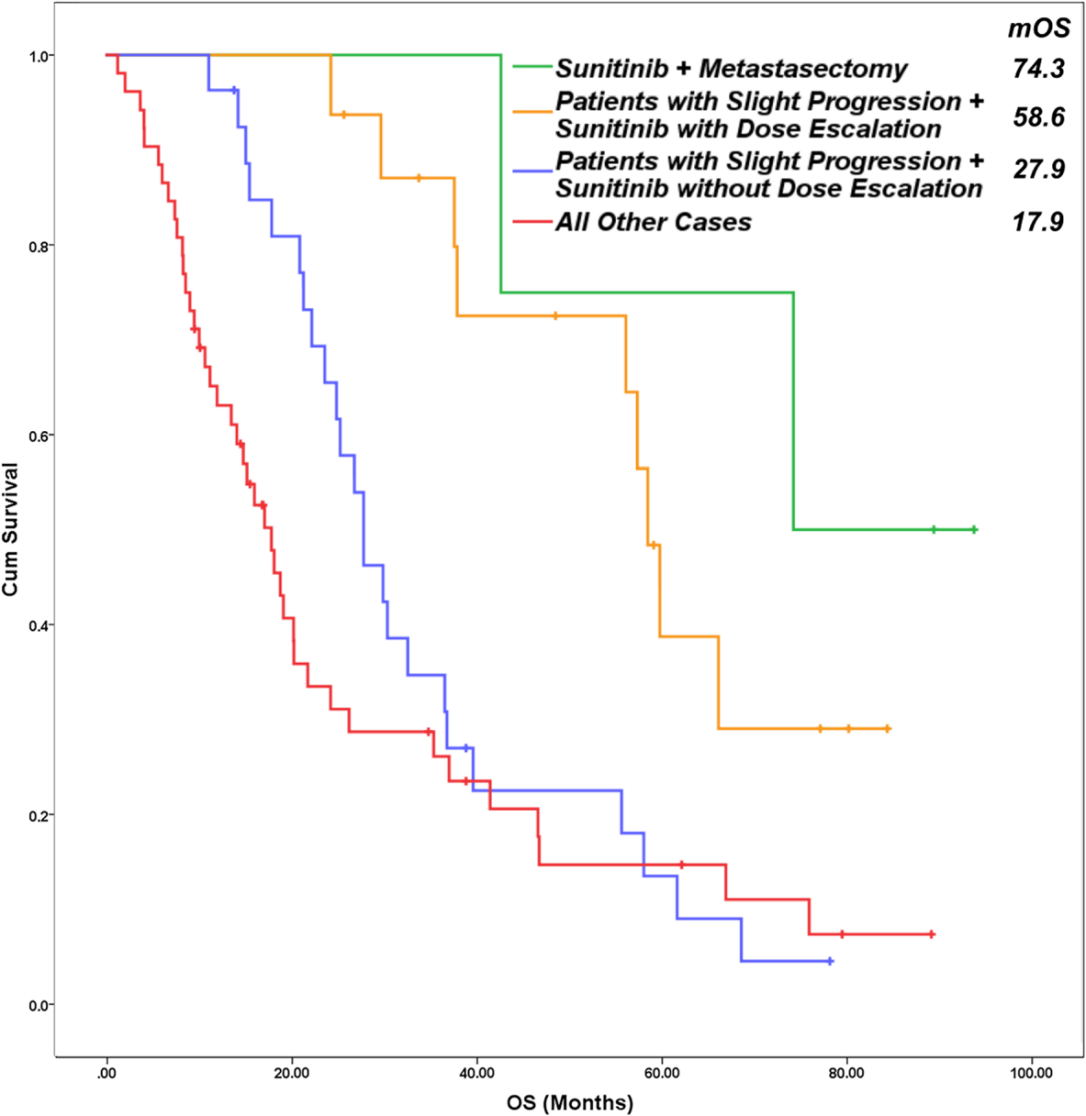
Overall survival of patients in four subgroups. Metastasectomy after an effective sunitinib therapy caused the most favorable overall survival (74.3 months). Median survival of patients with slight progression is longer with dose escalation (58.6 months) than without it (27.9 months), or the outcome of all other patients (17.9 months) (p‹0.001). (Cum – cumulative, OS – overall survival)

DE was performed in 18 (18.2%) cases among the evaluated 99 patients. PFS and OS results were more favorable when the dose was escalated rather than in case of patients without escalation. The dosing scheme was modified in 22 (22.2%) patients. If DSM was performed, the median PFS and OS were longer than without DSM. Dose escalation and DSM were independent parameters. The survival was longer as patients received more therapeutic lines after sunitinib treatment (Table 2) (Fig. 2).

The PFS and OS results of patients with SP who underwent radiological revision and showed to have a stable disease (48 patients), did not influence the number of metastatic sites, the MSKCC score, and the dose reduction. Age and gender of the patients did not influence the OS. PFS was longer in case of younger male patients. PFS and OS were more favorable if patients underwent nephrectomy, in case of DE and DSM (Table 3).

**Table 3:** Factors influencing the outcome of sunitinib therapy in SP cases

Specifications of all patients with slight progression *N* = 48	PFS-HR (95% CI)	*p*	OS-HR (95% CI)	***p***
Age	1.047 (1.008–1.089)	**0.019**	1.025 (0.982–1.069)	0.265
Number of metastatic organs	1.159 (0.873–1.538)	0.307	1.107 (0.820–1.494)	0.508
	PFS-HR (95% CI)	*p*	OS-HR (95% CI)	***p***
Gender				
man/woman	3.202 (1.473–6.962)	**0.003**	2.077 (0.891–4.846)	0.091
MSKCC score				
0 / 1 / 2	1 / 3.671 (0.474–28.414) / 5.304 (0.709–39.661)	0.176	1 / 2.965 (0.375–23.430) / 3.841 (0.513–28.786)	0.366
Dose reduction				
Yes / No	1 / 0.840 (0.450–1.570)	0.585	1 / 0.724 (0.365–1.436)	0.356
Nephrectomy				
Yes / No	1 / 3.397 (1.364–8.461)	**0.009**	1 / 5.583 (2.135–14.601)	**< 0.001**
Dose escalation				
Yes / No	1 / 2.383 (1.241–4.578)	**0.009**	1 / 2.479 (1.185–5.183)	**0.016**
Dose scheme modification				
Yes / No	1 / 2.373 (1.034–5.445)	**0.041**	1 / 2.583 (1.008–6.709)	**0.047**
Therapeutic lines after sunitinib				
2 / 1 / 0	NA	NA	1 / 6.163 (1.582–24.016) / 3.873 (1.130–13.280)	**0.032**

Bold *p*-values are significant ‹0.05, *HR* hazard ratio, *MSKCC* Memorial Sloan Kettering Cancer Center, *mOS* median overall survival, *mPFS* median progression-free survival, *NA* not applicable, *OS* overall survival, *p* p-value, *PFS* progression-free survival, *SE* standard error, *SP* slight progression

### Influence of dose escalation on effectivity

There were 23 patients in the control group (they underwent radiological revision before June 30, 2013 and showed slight progression) and 25 patients in the study group (they underwent radiological revision after June 30, 2013). The following factors were similar in the two groups: patients’ age, gender, MSKCC score, number of metastatic sites, time elapsed from diagnosis, serum calcium level, LDH, hemoglobin, Karnofsky performance status, DR and DSM. All patients underwent nephrectomy in the study group, whereas it was performed in 17 out of 23 patients in the control group (*p* = 0.008). Dose escalation was only performed in the study group. It could be performed in case of 18 patients (72.0%), but it could not be carried out in 7 cases (28.0%). Median PFS (39.7 ± 5.1 vs 14.2 ± 1.3 months (*p* = 0.037)) and mOS (57.5 ± 10.7 vs 27.9 ± 2.5 months (*p* = 0.044)) results were significantly better in the study group than in the control group (Table 4). The median follow-up time of the cohort with slight progression was 37.3 (11.17–93.83) months.

**Table 4:** Characteristics and results of patients with slight progression in the control and study groups

Specifications of patients with slight progression N_SP_ = 48	Control group Before June 30, 2013 N_CG_ = 23	Study group After June 30, 2013 N_SG_ = 25	*p*
Mean age, years ± SE	62.87 ± 1.73	60.74 ± 1.52	0.358
Gender			
male	17 (73.9%)	22 (88.0%)	0.190
female	6 (26.1%)	3 (12.0%)	
MSKCC score, mean ± SE	1.61 ± 0.1	1.60 ± 0.1	0.952
Number of metastatic sites, mean ± SE	2.17 ± 0.24	2.36 ± 0.21	0.559
*Location of metastases*			
Lungs	19 (82.6%)	20 (80%)	0.556
Bone	7 (30.4%)	9 (36%)	0.460
Distant lymph node	8 (34.8%)	12 (48%)	0.263
Liver	3 (13%)	4 (16%)	0.549
Suprarenal gland	1 (4.3%)	3 (12%)	0.337
Comorbidities			
Hypertension	4 (17.4%)	5 (20%)	0.556
Other cardiovascular disorders	2 (8.7%)	3 (12%)	0.541
Diabetes	2 (8.7%)	2 (8%)	0.663
Secondary tumors	0	1	0.521
Nephrectomy			
No	6 (26.1%)	0 (0.0%)	**0.008**
Yes	17 (73.9%)	25 (100.0%)	
Time from diagnosis to initiation of sunitinib			
< 1 year	11 (47.8%)	15 (60.0%)	0.289
> 1 year	12 (52.2%)	10 (40.0%)	
Hemoglobin level			
< normal range	6 (26.1%)	3 (12.0%)	0.190
> normal range	17 (73.9%)	22 (88.0%)	
Elevated corrected calcium level			
> 2.5 mmol/L	2 (8.7%)	1 (4.0%)	0.468
< 2.5 mmol/L	21 (91.3%)	24 (96.0%)	
Elevated LDH level			
> 1.5× normal level	2 (8.7%)	0 (0.0%)	0.224
< 1.5× normal level	21 (91.3%)	25 (100.0%)	
Elevated corrected calcium level			
> 2.5 mmol/L	2 (8.7%)	1 (4.0%)	0.468
< 2.5 mmol/L	21 (91.3%)	24 (96.0%)	
Karnofsky performance status			
< 80	0 (0.0%)	1 (4.0%)	0.521
≥ 80	23 (100.0%)	24 (96.0%)	
Dose reduction rate			
No	10 (43.5%)	16 (64.0%)	0.226
Level 1 (37.5 mg)	11 (47.8%)	6 (24.0%)	
Level 2 (25 mg)	2 (8.7%)	3 (12.0%)	
Dose escalation rate			
No	23 (100.0%)	7 (28.0%)	**< 0.001**
Level 1 (62.5 mg)	0 (0.0%)	14 (56.0%)	
Level 2 (75 mg)	0 (0.0%)	4 (16.0%)	
Dosing scheme modification			
No	19 (82.6%)	18 (72.0%)	0.300
Yes	4 (17.4%)	7 (28.0%)	
Therapeutic lines after sunitinib 0 / 1 / 2 (%)	6 (30) / 13 (65) / 1 (5)	3 (15) / 13 (65) / 4 (20)	0.247
mOS after sunitinib therapy	9.33 ± 2.0	9.76 ± 2.5	0.599
mPFS	14.2 ± 1.3	39.7 ± 5.1	**0.037**
mOS	27.9 ± 2.5	57.5 ± 10.7	**0.044**
median follow-up time (range) (months)	30.9 (11.2–89.5)	45.7 (13.9–84.5)	**0.061**

Bold *p*-values are significant ‹0.05, *mOS* median overall survival, *mPFS* median progression-free survival, *MSKCC* Memorial Sloan Kettering Cancer Center, *N* number of analyzed patients, *p* p-value, *SE* standard error, *SP* slight progression

Because of the higher rate of nephrectomy and DE in study group, a multivariate analysis was performed to detect the real effect of these factors. Based on a multivariate COX analysis, both DE (HR_DE_: 2.12, 95% CI 1.077–4.181; *p*_*DE*_ = 0.030) and nephrectomy (HR_nephr._: 2.47, 95% CI 1.023–6.315; *p*_*nephr.*_ = 0.049) were independent factors of PFS in patients with SP. In relation to OS, only nephrectomy influenced the results independently (HR_nephr._: 5.02, 95% CI 1.94–12.98; *p*_*nephr.*_ = 0.001) but DE did not (*p*_*DE*_ = 0.083).

### The impact of dose escalation on the adverse effects

After dose escalation, the most common adverse effects were the following: worsening or development of fatigue, hypertension, stomatitis, and weight loss (over 10%) (Table 5). The most upgraded clinical parameters were fatigue and development or worsening of hypertension as a result of the increased sunitinib dose.

**Table 5:** New or intensifying adverse effects in patients after dose escalation

New or intensifying adverse effects N_DE_ = 22	Number of patients (percent)			
	Any grade	Grade 1	Grade 2	Grade 3
All	21 (95.5%)	17 (77.3%)	3 (13.6%)	1 (4.5%)
Fatigue	9 (40.9%)	7 (31.8%)	2 (9.1%)	0
Development / worsening of hypertension	8 (36.4%)	7 (31.8%)	1 (4.5%)	0
Stomatitis	6 (27.3%)	5 (22.7%)	1 (4.5%)	0
Diarrhea	5 (22.7%)	3 (13.6%)	1 (4.5%)	1 (4.5%)
Weight loss 10%≤	4 (18.2%)	4 (18.2%)	0	0
Hand–foot syndrome	4 (18.2%)	4 (18.2%)	0	0
Eyelid edema	2 (9.1%)	2 (9.1%)	0	0
Hypothyroidism	1 (4.5%)	1 (4.5%)	0	0
Elevation in creatinine level	5 (18.2%)	4 (18.2%)	1 (4.5%)	0
Thrombocytopenia	4 (18.2%)	2 (9.1%)	2 (9.1%)	0
Anemia	3 (13.6%)	2 (9.1%)	1 (4.5%)	0
Neutropenia	2 (9.1%)	1 (4.5%)	1 (4.5%)	0

## Discussion

Sunitinib is one of the most frequently applied first line therapies in patients with metastatic ccRCC with MSKCC good and moderate prognoses.

The role of cytoreductive nephrectomy seems to be equivocal in the era of tyrosine-kinase inhibition. The results of the SURTIME study were presented by Bex et al. last year, in which the overall survival and post surgical complication rates were better with deferred versus immediate cytoreductive nephrectomy, while progression rates at 16 and 28 weeks were not significantly different between both sequences [32]. The ongoing CARMENA study (NCT00930033) may give an answer to this issue in the near future.

According to the recent knowledge, nephrectomy is recommended to be performed in patients in good general condition before the systemic therapy; however, randomized studies analyzing survival data have been performed only in combination with INFα therapy [33–35]. In our study, nephrectomy was performed in 84.8% of the cases, and PFS and OS results of these patients were more favorable. Each patient with SP in the Study group (period 2) underwent nephrectomy (which means that the patients were fit enough for this operation). It might have been a potential selectional bias of the compared cohorts. However, the other parameters and the comorbidities of the patients in the two cohorts were not significantly different.

In our study, PFS was longer than in the registration study [8]; however, patients with MSKCC poor prognosis were excluded from our study, but the PFS of our patients was similar to the excellent international data [36, 37]. Nowadays, the median OS of patients with metastatic RCC is longer than 2 years [1], as it can be seen in our results as well.

One of the most important things in case of a successfully optimized medical therapy is appropriate dosing: the individually titrated, tolerable dose, with the administration of the maximum daily dose. It is important to choose the most suitable dosing scheme after taking comorbidities into consideration [38]. The recommended starting dose for sunitinib malate is 50 mg daily for 28 days followed by a 14-day break. Although individualized sunitinib therapy improves the outcome, poorer outcomes in patients tolerating the standard schedule treatment without significant toxicity [1, 14] may be the result of underdosing [27]. Several authors [39, 40] have reported that both PFS and OS are significantly higher in patients with at least grade 2 hypertension. As on-target side effects determine the drug effect, toxicity profile can be used to optimize dosing and treatment schedules individually [41]. According to the meta-analysis of Houk et al. [28], escalated sunitinib exposure (area under the curve) is associated with improved clinical outcomes as well as with an increased risk of adverse effects. The appropriate management of adverse events is necessary for effective sunitinib treatment, which requires the active contribution of the satisfactorily informed patient. Based on the above mentioned data, dose escalation has been applied after the summer of 2013 in cases with slight progression, when RECIST 1.1 results confirmed a stable disease if any clinically relevant side effects occurred. Our idea was to achieve the optimal titration of sunitinib until the appearance of on target side effects depending on the tolerable off target adverse events. The rate of CR according to RECIST in our studied population was relatively high (7.1%) compared to pivotal phase III trials of sunitinib [8], which might reflect an outstanding benefit from sunitinib mainly in patients with low tumor volume in our studied cohort. After an initial favor tumor response evolving slight progression can be stopped or be reversible with dose escalation and adequate titration has been hypothesized. Drug toxicity and efficacy may depend on the interindividual differences in pharmacokinetics, pharmacodynamics, and pharmacogenetics [42, 43]; however, Motzer et al. [14] have not found correlation between sunitinib pharmacokinetic values and the toxicity profile. Adelaiye et al. [26] have detected an increase in sunitinib plasma concentration in animals treated with escalated dose TKI in the drug resistant group, and also a trend for decreased plasma concentration after prolonged sunitinib exposure. Gotink et al. [24] have found 1.7 to 2.5-fold increase in sunitinib concentration in resistant tumor cells due to the increased lysosomal drug sequestration, which was reversible after the removal of sunitinib from the cell culture. Blood levels of sunitinib reach a steady state at 10 to 14 days, and a maximum value on day 14 [27], and disease progression usually occurs during treatment interruption [44, 45]. In the retrospective analysis of Bjarnason et al. [27], an individualized treatment strategy and shorter treatment break (14 days on and 7 days off) have resulted in improved PFS and OS as compared to the standard sunitinib schedule, and the PFS detected in patients with ccRCC has been one of the best reported for any TKI. Modified sunitinib schedule is well tolerated and induces optimal drug exposure [46].

Based on our results, PFS and OS results can be improved by sunitinib dose escalation as by dose scheme modification in case of patients poorly tolerating the therapy. As the two patient populations are not the same, their effects can be considered independent. Dose escalation can be performed in case of patients with good general condition, who do not have any relevant adverse effects. In case of these patients, based on the prognostic values, the survival rate is potentially better. Therefore, we compared the two (almost similar) groups regarding dose escalation, so selection of patients with better prognosis could not have queried the results. The effect of dose escalation on PFS and OS was confirmed during the comparison of the two groups. No significant difference was found among the number of the subsequent therapies and mOS after sunitinib was equal in two groups as well, which may be because in our country the availability of more active new regimens was very limited during our study period.

The rate of adverse events (AE) in our real world dose escalated patients is lower in the selected cohort than the AE rate in patients administered the standard dose in the pivotal trials [8, 9]. It might be partly explained by the favorable VEGFR inhibitor tolerability and the better proactive management of toxicity, which may improve the tolerability of the drug.

Acquired resistance to sunitinib therapy, driven by several likely mechanisms, is a central issue in the treatment of metastatic RCC patients. However, drug resistance may be reversible, and gradual dose escalation may restore tumor sensitivity to sunitinib, as reported in preclinical and clinical studies as well. Adelaiye et al. [26] have treated mice with patient-derived xenografts 5 days/week with a 40–60-80 mg/kg sunitinib dose increase schedule, and they have found selected intrapatient dose escalation safe, resulting in prolonged PFS due to a greater and longer effect on tumor regression. Although xenografts initially responsive to 40 mg/kg sunitinib developed drug resistance, it could be overcome by incremental dose escalation. In metastatic RCC patients on standard schedule sunitinib with early disease progression, Adelaiye et al. [26] could increase sunitinib dose from 50 to 62.5 and 75 mg daily, with a 14-day on and 7-day off treatment scheme to some type of grade 2 toxicity, and they observed clinical benefit in the majority of the patients. As reported by Mitchell et al. [47], the daily dose of sunitinib can be safely up-titrated to 87.5 mg. According to Gotink et al. [24] and Zama et al. [48], sunitinib rechallenging in previously resistant patients also has a therapeutic value. Drug resistance is also associated with epigenetic changes in histone proteins in the chromatin, which may be reversible upon DE; thus, epigenetic therapies could be successful in ccRCC patients [26].

The limitations of our study are, on the one hand, its retrospective design, that is, an explorative retrospective analysis of a prospective RCC register, and on the other hand, the relatively small number of patients involved.

## Conclusion

In conclusion, an individual escalated sunitinib therapy optimized by toxicity profile in metastatic RCC patients prolongs PFS and OS, and it is a safe treatment option with a moderate increase in adverse effects. Based on our data, dose escalation in 12.5 mg steps may be recommended for properly educated patients with slight progression, when RECIST 1.1 results confirm a stable disease in case any clinically relevant adverse effects occurred.

## Abbreviations

ATP: Adenosine triphosphate
bFGF: b-fibroblast growth factor
ccRCC: Clear cell renal cell carcinoma
CT: Computed tomography
DE: Dose escalation
DR: Dose reduction
DSM: Dose scheme modification
EAU: European Association of Urology
ESMO: European Society for Medical Oncology
HR: Hazard ratio
IL: Illinois
IL-8: Interleukin-8
LDH: Lactate dehydrogenase
LVEF: Left ventricular ejection fraction
mOS: Median overall survival
mPFS: Median progression-free survival
mRCC: Metastatic renal cell carcinoma
MSKCC: Memorial sloan kettering cancer center
NA: Not Applicable
NCCN: National Comprehensive Cancer Network
NCI CTCAE v4.0: National Cancer Institute Common Terminology Criteria for Adverse Events Version 4.0
ORR: Overall response rate
OS: Overall survival
PDGFRA: Platelet-derived growth factor receptor alpha
PDGFRB: Platelet-derived growth factor receptor beta
PFS: Progression-free survival
RCC: Renal cell carcinoma
RECIST: Response evaluation criteria in solid tumors
RTK: Receptor tyrosine kinase
SE: Standard error
SP: Slight progression
TKI: Tyrosine kinase inhibitor
USA: United States of America
VEGFR: Vascular endothelial growth factor receptor

## References

[1] Motzer RJ, Hutson TE, Tomczak P, Michaelson MD, Bukowski RM, Oudard S (2009). Overall survival and updated results for sunitinib compared with interferon alfa in patients with metastatic renal cell carcinoma. J Clin Oncol.

[2] Sun L, Liang C, Shirazian S, Zhou Y, Miller T, Cui J (2003). Discovery of 5-[5-fluoro-2-oxo-1,2- dihydroindol-(3Z)-ylidenemethyl]-2,4- dimethyl-1H-pyrrole-3-carboxylic acid (2-diethylaminoethyl)amide, a novel tyrosine kinase inhibitor targeting vascular endothelial and platelet-derived growth factor receptor tyrosine kinase. J Med Chem.

[3] Mendel DB, Laird AD, Xin X, Louie SG, Christensen JG, Li G (2003). In vivo antitumor activity of SU11248, a novel tyrosine kinase inhibitor targeting vascular endothelial growth factor and platelet-derived growth factor receptors: determination of a pharmacokinetic/pharmacodynamic relationship. Clin Cancer Res.

[4] O'Farrell AM, Abrams TJ, Yuen HA, Ngai TJ, Louie SG, Yee KW (2003). SU11248 is a novel FLT3 tyrosine kinase inhibitor with potent activity in vitro and in vivo. Blood.

[5] Abrams TJ, Lee LB, Murray LJ, Pryer NK, Cherrington JM (2003). SU11248 inhibits KIT and platelet-derived growth factor receptor beta in preclinical models of human small cell lung cancer. Mol Cancer Ther.

[6] Potapova O, Laird AD, Nannini MA, Barone A, Li G, Moss KG (2006). Contribution of individual targets to the antitumor efficacy of the multitargeted receptor tyrosine kinase inhibitor SU11248. Mol Cancer Ther.

[7] Chow LQ, Eckhardt SG (2007). Sunitinib: from rational design to clinical efficacy. J Clin Oncol.

[8] Motzer RJ, Hutson TE, Tomczak P, Michaelson MD, Bukowski RM, Rixe O (2007). Sunitinib versus interferon alfa in metastatic renal-cell carcinoma. N Engl J Med.

[9] Cella D, Michaelson MD, Bushmakin AG, Cappelleri JC, Charbonneau C, Kim ST (2010). Health-related quality of life in patients with metastatic renal cell carcinoma treated with sunitinib vs interferon-alpha in a phase III trial: final results and geographical analysis. Br J Cancer.

[10] NCCN Guideline: https://www.nccn.org/professionals/physician_gls/pdf/kidney.pdf. Accessed 6 Feb 2018.

[11] Escudier B, Porta C, Schmidinger M, Rioux-Leclercq N, Bex A, Khoo V (2016). Renal Cell Carcinoma: ESMO Clinical Practice Guidelines. Ann Oncol.

[12] Ljungberg B, Bensalah K, Canfield S, Dabestani S, Hofmann F, Hora M (2015). EAU Guidelines on Renal Cell Carcinoma: 2014 Update. Eur Urol.

[13] Schmidinger M, Larkin J, Ravaud A (2012). Experience with sunitinib in the treatment of metastatic renal cell carcinoma. Ther Adv Urol.

[14] Motzer RJ, Hutson TE, Olsen MR, Hudes GR, Burke JM, Edenfield WJ (2012). Randomized phase II trial of sunitinib on an intermittent versus continuous dosing schedule as first-line therapy for advanced renal cell carcinoma. J Clin Oncol.

[15] Barrios CH, Hernandez-Barajas D, Brown MP, Lee SH, Fein L, Liu JH (2012). Phase II trial of continuous once-daily dosing of sunitinib as first-line treatment in patients with metastatic renal cell carcinoma. Cancer.

[16] Bracarda S, Iacovelli R, Boni L, Rizzo M, Derosa L, Rossi M (2016). Sunitinib administered on 2/1 schedule in patients with metastatic renal cell carcinoma: the RAINBOW analysis. Ann Oncol.

[17] Escudier B. ESMO 2017: Nivolumab Plus Ipilimumab versus Sunitinib in First-Line Treatment for Advanced or Metastatic RCC. http://www.esmo.org/Conferences/ESMO-2017-Congress/News-Articles/Nivolumab-Plus-Ipilimumab-versus-Sunitinib-in-First-Line-Treatment-for-Advanced-or-Metastatic-RCC. Accessed 10 Sept 2017.

[18] Sutent SMPC: http://www.ema.europa.eu/docs/en_GB/document_library/EPAR_-_Summary_for_the_public/human/000687/WC500057689.pdf. Accessed 3 Oct 2014.

[19] Kerbel RS (2001). Molecular and physiologic mechanisms of drug resistance in cancer: an overview. Cancer Metastasis Rev.

[20] Kerbel RS, Yu J, Tran J, Man S, Viloria-Petit A, Klement G (2001). Possible mechanisms of acquired resistance to anti-angiogenic drugs: implications for the use of combination therapy approaches. Cancer Metastasis Rev.

[21] Ellis LM, Hicklin DJ (2008). VEGF-targeted therapy: mechanisms of anti-tumour activity. Nat Rev Cancer.

[22] Bottsford-Miller JN, Coleman RL, Sood AK (2012). Resistance and escape from antiangiogenesis therapy: clinical implications and future strategies. J Clin Oncol.

[23] Gerber PA, Hippe A, Buhren BA, Muller A, Homey B (2009). Chemokines in tumor associated angiogenesis. Biol Chem.

[24] Gotink KJ, Broxterman HJ, Labots M, de Haas RR, Dekker H, Honeywell RJ (2011). Lysosomal sequestration of sunitinib: a novel mechanism of drug resistance. Clin Cancer Res.

[25] Hammers HJ, Verheul HM, Salumbides B, Sharma R, Rudek R, Jaspers J (2010). Reversible epithelial to mesenchymal transition and acquired resistance to sunitinib in patients with renal cell carcinoma: evidence from a xenograft study. Mol Cancer Ther.

[26] Adelaiye R, Ciamporcero E, Miles KM, Sotomayor P, Bard J, Tsompana M (2015). Sunitinib dose-escalation overcomes transient resistance in clear cell renal cell carcinoma and is associated with epigenetic modifications. Mol Cancer Ther.

[27] Bjarnason GA, Khalil B, Hudson JM, Williams R, Milot LM, Atri M (2014). Outcomes in patients with metastatic renal cell cancer treated with individualized sunitinib therapy: correlation with dynamic microbubble ultrasound data and review of the literature. Urol Oncol.

[28] Houk BE, Bello CL, Poland B, Rosen LS, Demetri GD, Motzer RJ (2010). Relationship between exposure to sunitinib and efficacy and tolerability endpoints in patients with cancer: results of a pharmacokinetic/pharmacodynamic meta-analysis. Cancer Chemother Pharmacol.

[29] NCI CTCAE https://www.eortc.be/services/doc/ctc/CTCAE_4.03_2010-06-14_QuickReference_5x7.pdf. Accessed 14 June 2010.

[30] Schag CC, Heinrich RL, Ganz PA (1984). Karnofsky performance status revisited: reliability, validity, and guidelines. J Clin Oncol.

[31] Eisenhauer EA, Therasse P, Bogaerts J, Schwartz LH, Sargent D, Ford R (2009). New response evaluation criteria in solid tumours: revised RECIST guideline (version 1.1). Eur J Cancer.

[32] Bex A, Mulders P, Jewett M, et al. Immediate versus deferred cytoreductive nephrectomy (CN) in patients with synchronous metastatic renal cell carcinoma (mRCC) receiving sunitinib (EORTC 30073 SURTIME). Abstract presented at: ESMO 2017 Congress; September 8–12, 2017; Madrid, Spain. Abstract LBA35.

[33] Mickisch GH, Garin A, van Poppel H, de Prijck L, Sylvester R, European Organisation for Research and Treatment of Cancer (EORTC) Genitourinary Group (2001). Radical nephrectomy plus interferon-alfa-based immunotherapy compared with interferon alfa alone in metastatic renal-cell carcinoma: a randomised trial. Lancet.

[34] Jonasch E, Haluska FG (2001). Interferon in oncological practice: review of interferon biology, clinical applications, and toxicities. Oncologist.

[35] Aslam MZ, Matthews PN (2014). Cytoreductive nephrectomy for metastatic renal cell carcinoma: a review of the historical literature and its role in the era of targeted molecular therapy. ISRN Urol.

[36] Rautiola J, Donskov F, Peltola K, Joensuu H, Bono P. Sunitinib-induced hypertension, neutropaenia and thrombocytopaenia as predictors of good prognosis in patients with metastatic renal cell carcinoma. BJU Int. 2016;117(1):110–7. 10.1111/bju.12940 25252180

[37] Kucharz J, Dumnicka P, Kuzniewski M, Kusnierz-Cabala B, Herman RM, Krzemieniecki K (2015). Co-occurring adverse events enable early prediction of progression-free survival in metastatic renal cell carcinoma patients treated with sunitinib: a hypothesis-generating study. Tumori.

[38] Schmidinger M, Arnold D, Szczylik C, Wagstaff J, Ravaud A (2010). Optimizing the use of sunitinib in metastatic renal cell carcinoma: an update from clinical practice. Cancer Investig.

[39] Rini BI, Cohen DP, Lu DR, Chen I, Hariharan S, Gore ME (2011). Hypertension as a biomarker of efficacy in patients with metastatic renal cell carcinoma treated with sunitinib. J Natl Cancer Inst.

[40] Donskov F, Michaelson MD, Puzanov I, Davis MP, Bjarnason GA, Motzer RJ (2015). Sunitinib-associated hypertension and neutropenia as efficacy biomarkers in metastatic renal cell carcinoma patients. Br J Cancer.

[41] Rini BI, Melichar B, Ueda T, Grünwald V, Fishman MN, Arranz JA (2013). Axitinib with or without dose titration for first-line metastatic renal-cell carcinoma: a randomised double-blind phase 2 trial. Lancet Oncol.

[42] Klumpen HJ, Samer CF, Mathijssen RH, Schellens JH, Gurney H (2011). Moving towards dose individualization of tyrosine kinase inhibitors. Cancer Treat Rev.

[43] Houk BE, Bello CL, Kang D, Amantea M (2009). A population pharmaco- kinetic meta-analysis of sunitinib malate (SU11248) and its primary metabolite (SU12662) in healthy volunteers and oncology patients. Clin Cancer Res.

[44] Mancuso MR, Davis R, Norberg SM, O'Brien S, Sennino B, Nakahara T (2006). Rapid vascular regrowth in tumors after reversal of VEGF inhibition. J Clin Invest.

[45] Motzer RJ, Michaelson MD, Redman BG, Hudes GR, Wilding G, Figlin RA (2006). Activity of SU11248, a multitargeted inhibitor of vascular endothelial growth factor receptor and platelet-derived growth factor receptor, in patients with metastatic renal cell carcinoma. J Clin Oncol.

[46] Faivre S, Delbaldo C, Vera K, Robert C, Lozahic S, Lassau N (2006). Safety, pharmacokinetic, and antitumor activity of SU 11248, a novel oral multitarget tyrosine kinase inhibitor, in patients with cancer. J Clin Oncol.

[47] Mitchell N, Fong PC, Broom RJ (2015). Clinical experience with sunitinib dose escalation in metastatic renal cell carcinoma. Asia Pac J Clin Oncol.

[48] Zama IN, Hutson TE, Elson P, Cleary JM, Choueiri TK, Heng DY (2010). Sunitinib rechallenge in metastatic renal cell carcinoma patients. Cancer.

